# Hinokiflavone induces apoptosis and inhibits migration of breast cancer cells via EMT signalling pathway

**DOI:** 10.1002/cbf.3443

**Published:** 2020-02-27

**Authors:** Wenzhen Huang, Chi Liu, Fengen Liu, Zhiyong Liu, Guie Lai, Jian Yi

**Affiliations:** ^1^ Department of Vascular and Breast Surgery First Affiliated Hospital of Gannan Medical University Ganzhou Jiangxi P.R. China; ^2^ School of Medical & Life Sciences Chengdu University of TCM Chengdu Sichuan P.R. China

**Keywords:** hinokiflavone, breast cancer, apoptosis, migration, epithelial‐to‐mesenchymal transition

## Abstract

Hinokiflavone is a natural product, isolated from *Selaginella* P. Beauv, *Juniperus phoenicea* and *Rhus succedanea*. Even though hinokiflavone was reported to possess cytotoxicity to many cancer cells, and has potential in cancer treatment, the anti‐proliferation and anti‐metastasis efficacy of hinokiflavone on human breast cancer cells has not a further research. In this study, we investigated the anti‐cancer activity of hinokiflavone in human breast cancer cells *in vitro* and *in vivo*. Hinokiflavone exhibited a time‐ and dose‐dependent manner apoptosis induction by upregulating expression of Bax and downregulating Bcl‐2 in breast cancer cells. Furthermore, hinokiflavone significantly inhibited the migration and invasion of breast cancer cells by impairing the process of epithelial‐to‐mesenchymal transition. In addition, the tumour growth was distinctly inhibited by treatment of hinokiflavone in a xenograft tumour mouse model of MDA‐MB‐231 cells. Immunohistochemical analysis of tumour sections showed that MMP‐2^+^ cells and Ki‐67^+^ cells were remarkably decreased in tumour tissues of mice after treatment of hinokiflavone, indicating that hinokiflavone inhibits not only proliferation but also metastasis of breast cancer cells. Our study suggested that hinokiflavone can be a potential drug to breast cancer.

**Significance of the study:**

Hinokiflavone significantly inhibited proliferation and induced apoptosis in breast cancer cells. In addition, hinokiflavone remarkably inhibited migration and invasion of breast cancer cells via EMT signalling pathway. It is worth noting that hinokiflavone possesses anti‐tumour effect in tumour mouse xenograft model of breast cancer. Overall, our results indicated that hinokiflavone may be a potential anticancer drug for breast cancer treatment.

## 1 INTRODUCTION

According to statistics, the three most commonly diagnosed types of cancer in women were breast, lung and colorectum in the USA in 2015. In particular, breast cancer, alone is expected to account for approximately 30% of all yearly diagnosed cancer cases in women.^1^ In addition, breast cancer, like most cancers, is on the rise in developing countries such as Brazil and China.^2^ Although breast cancer has made some progress, the great threat to women from this cancer is still very serious, and particularly the patients who suffer from the ‘triple‐negative’ breast cancer (TNBC), which is negative for the expressions of progesterone (PR), estrogen receptors (ER) and HER2.^3^, ^4^, ^5^ Compared with non‐TNBC, advanced TNBC has aggressive clinical progress and poor prognosis.^6^ Moreover, highly malignant and metastatic of breast cancer is a principle cause of female mortality.^7^ Unfortunately, there is no effective therapeutic to control the recurrence and metastasis of breast cancer. Thus, it is necessary to search for effective drug candidates with potential anti‐tumour activity and low toxicity for metastatic breast cancer.

Natural products have been used to treat human diseases for thousands of years and are of great value in the discovery and development of drugs.^8^, ^9^ Many anti‐cancer and anti‐infectious agents are derived from natural products.^9^ Furthermore, in recent decades, the rapid development of more effective drugs with fewer side effects has been a common goal for scientists and clinicians.^10^ Because of its low side effects, it is crucial to identify natural disease‐resistant plant compounds from medicinal plants and natural products.^11^

**Figure 1:**
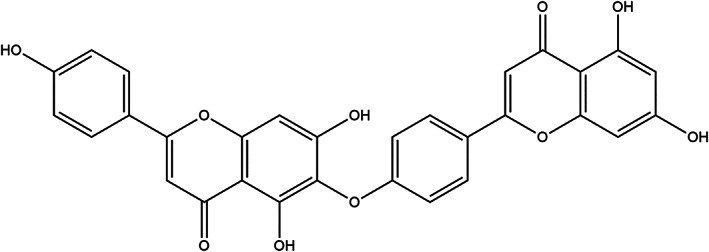
Chemical structure of hinokiflavone

Hinokiflavone (Figure 1), a natural product, derived from several plants such as *Selaginella* P. Beauv, *Juniperus phoenicea* and *Rhus succedanea* and so on,^12^, ^13^ has proved to have several biological activities, including anti‐HIV‐1, reverse transcriptase,^14^ antiinfluenza virus sialic acid enzyme^15^ and antioxidant activity.^16^ Lin *et al*. reported that hinokiflavone exhibited antitumour activity in KB human oral cancer cells *in vitro*.^17^ Sukesh Kalva *et al*. proved that hinokiflavone had a good suppressed activity against MMP2 and MMP9.^18^ MMP2 and MMP9, affiliated to the matrix metalloproteinase (MMP) family, were regarded as attractive targets for various cancer therapies, which was involved in the tumour metastasis, growth and neovascularization.^19^, ^20^, ^21^, ^22^ However, the effects of hinokiflavone on inhibiting pulmonary metastasis of breast cancer and its related molecular mechanism have not yet been reported. Considering the effects of hinokiflavone on MMPs, we hypothesized that hinokiflavone might inhibit the migration and invasion of breast cancer. Therefore, the anti‐proliferation and anti‐metastasis efficacies of hinokiflavone *in vitro* and *in vivo* were assessed in breast cancer cells in the current study. Furthermore, the anti‐metastasis mechanism of hinokiflavone was also explored.

## 2 MATERIALS AND METHODS

### 2.1 Reagents

Hinokiflavone as a 98% purity that detected by HPLC was bought from Chengdu Biopurify Phytochemicals Ltd (Chengdu, China). 3‐(4,5‐Dimethylthiazol‐2‐yl)‐2,5‐diphenyltetrazolium bromide (MTT) was purchased from Sigma Chemical Co. (St Louis, MO, USA). The Annexin VFITC and PI Apoptosis Detection Kit and Hoechst 33258 were bought from KeyGen Biotech (Nanjing, China). The primary antibodies against Bax, Bcl‐2, caspase‐3, cleavedcaspase‐3, β‐actin, E‐cadherin and N‐cadherin were purchased from Cell Signaling Technology (Beverly, MA, USA) for western blot experiments. Secondary antibodies were supplied by ZSGB‐BIO Co. (Beijing, China).

The stock solution of hinokiflavone (40 mM) which was dissolved in DMSO was stored at −20 °C. The stock solution was diluted with culture medium for further experimental use.

### 2.2 Cell culture

The human triple negative breast cancer cell line of MDA‐MB‐231 and mouse triple negative breast cancer cell line of 4T1 were purchased from American Type Culture Collection (ATCC, Rockville, MD, USA). Dulbecco's Modified Eagle Medium (DMEM) containing 1% antibiotics (penicillin and streptomycin) and 10% heat in activated foetal bovine serum (FBS, Gibco, Auckland, N.Z.) was applied to culture cell in atmosphere of 5% CO_2_ and 37 °C.

### 2.3 Cell proliferation assay

Cells were seeded in the 96‐well plates and exposed with different concentrations (0, 0.625, 1.25, 2.5, 5, 10, 20 and 40 μM) of hinokiflavone. After incubation for 24, 48 and 72 h, 20 μl of MTT solution (5 mg ml^−1^) was added to each well. Cells were continuously incubated for another 4 h. The purple formazan crystal which was dissolved in 150 μl of DMSO was quantified by measurement of the absorbance at 570 nm using a Spectra MAX M5 micro plate spectrophotometer (Molecular Devices, CA, USA). Data were showed as means ± SD of at least three independent experiments.

### 2.4 Colony formation assay

Colony formation assay was conducted according to guideline. In short, MDA‐MB‐231 and 4T1 cells were seeded in six‐well plates at 200–500 cells/well respectively. After 24 h incubation, cells were exposed to various concentrations (0, 1.25, 2.5, 5, 10 and 20 μM) of hinokiflavone for 9 days. After that, cells were washed with phosphate‐buffered saline (PBS, pH 7.4) three times, fixed with methanol for 20 min and stained with 0.5% crystal violet solution for 15 min. Colonies (> 50 cells) were counted under the microscope. Data was showed as the average of three independent experiments.

### 2.5 Cell‐apoptosis analysis

Cell apoptosis was assessed by flow cytometry (FCM) assay. In brief, cells were treated with determined concentrations of hinokiflavone (0, 10, 20 and 40 μM) for 48 hours. Then, cells were harvested, washed with ice‐cold PBS and stained with annexin V‐FITC/PI dual staining apoptosis detection kits (BD Biosciences, San Diego, CA). The apoptotic cells were quantified using a flow cytometer (BD Biosciences).

### 2.6 Western blot analysis

The western blot assay was conducted as follows. In brief, 4T1 and MDA‐MB‐231 cells which were treated with hinokiflavone in different concentrations for 48 h, were harvested, washed twice with cold PBS and lysed in RIPA buffer. The concentrations of total protein were measured by the Lowry method. Equal amounts of protein from each sample were loaded and separated by sodium dodecyl sulfate‐polyacrylamide gel electrophoresed gels and transferred onto polyvinylidene difluoride membranes (Amersham Bioscience, Piscataway, NJ, USA). Then, the membranes were blocked by nonfat milk for 2 h at 37 °C and incubated with specific primary antibodies overnight at 4 °C. After incubation with the corresponding secondary antibodies, the targeted protein bands were visualized using an enhanced chemiluminescence kit (Amersham).

### 2.7 Wound‐healing migration assay

Wound‐healing migration assay was performed as follows. Cancer cells which grew to about 90% confluence were scraped by sterile 0.1 ml pipette tips, and were cultured in fresh medium contains only 1% FBS and different concentrations of hinokiflavone. After 48 h incubation, cells were washed, fixed and photographed. Images were obtained using a microscope (Zeiss, Jena, Germany), and the inhibited percentage of migrated cells was showed using 100% as the specific value referred to untreated group.

### 2.8 Boyden chamber migration and invasion assay

Boyden chamber (8 μm pore size) migration and invasion assays were performed as follows. In brief, a total of 5×10^4^ cells (for MDA‐MB‐231) which were suspended in 100μl serum‐free cell culture medium were added in the upper chamber, and 600μl of cell culture medium containing 10% FBS was added at the bottom wells of plate. Different concentrations of hinokiflavone were added in upper chambers. Cells were allowed to migrate for 24 and 48 h. Nonmigrated cells which were located on the upper chamber were removed using a cotton swab. The migrated cells were fixed in methanol and stained with 0.5% crystal violet. Migrated cells were imaged and counted in six randomly selected fields by using a light microscope. As to invasion assay, cells which were suspended in 100μl serum‐free cell culture medium were added in the upper chamber which were pre‐coated with serum‐free medium diluted Matrigel (1:3, 60 μl well^−1^, BD Biosciences), and 600 μl medium with 10% FBS was added to the lower compartment of the chambers. After treated with different concentrations of hinokiflavone for 48 h, cells which were located on the upper chamber were removed using a cotton swab. Cells located on the underside of the filter were fixed with methanol and stained with 0.5% crystal violet. Invasive cells were imaged and counted in six randomly selected fields by using a light microscope. The results were showed as the inhibited percentage rate of migration/invasion compared with untreated group.

### 2.9 Mice and tumour model

Female Balb/c nude mice (6–8 weeks old) were bought from HFK bioscience CO., LTD, Beijing, China. All animal experiments were approved by the Ethics Committee and Institutional Animal Care and Treatment Committee of First Affiliated Hospital of Gannan Medical University. Mice were inoculated with 100 μl ofMDA‐MB‐231 cell suspension (1.0×10^7^ cells). At 5 days after inoculation, when the tumour volume had reached about 100 mm^3^, the mice were equally and randomly divided into three treatment groups (control, 20 mg kg^−1^ and 40 mg kg^−1^) (*n*=6). Mice received hinokiflavone treatment by intraperitoneal injection once daily for three weeks. Tumour volumes and body weight were measured every 3 days. The tumour size was calculated according to the formula: Tumour volume (mm^3^) = 0.5 × L × W^2^, where L is the length and W is the width. At the termination of the experiment, all animals were euthanized by cervical dislocation. The tumours were isolated, imaged, weighted and fixed with paraformaldehyde for further immunohistochemistry evaluation.

### 2.10 Statistical analysis

All experiments were performed at least in triplicate. Data were represented as mean ± SD of three independent experiments. *P*‐values for comparison of two groups were determined by two‐tailed Student's *t*‐test. Statistically significant *P*‐values were labelled as follows: *P <0.05; **P <0.01; ***P <0.001.

## 3 RESULTS

### 3.1 Hinokiflavone inhibits melanoma cell proliferation

**Figure 2:**
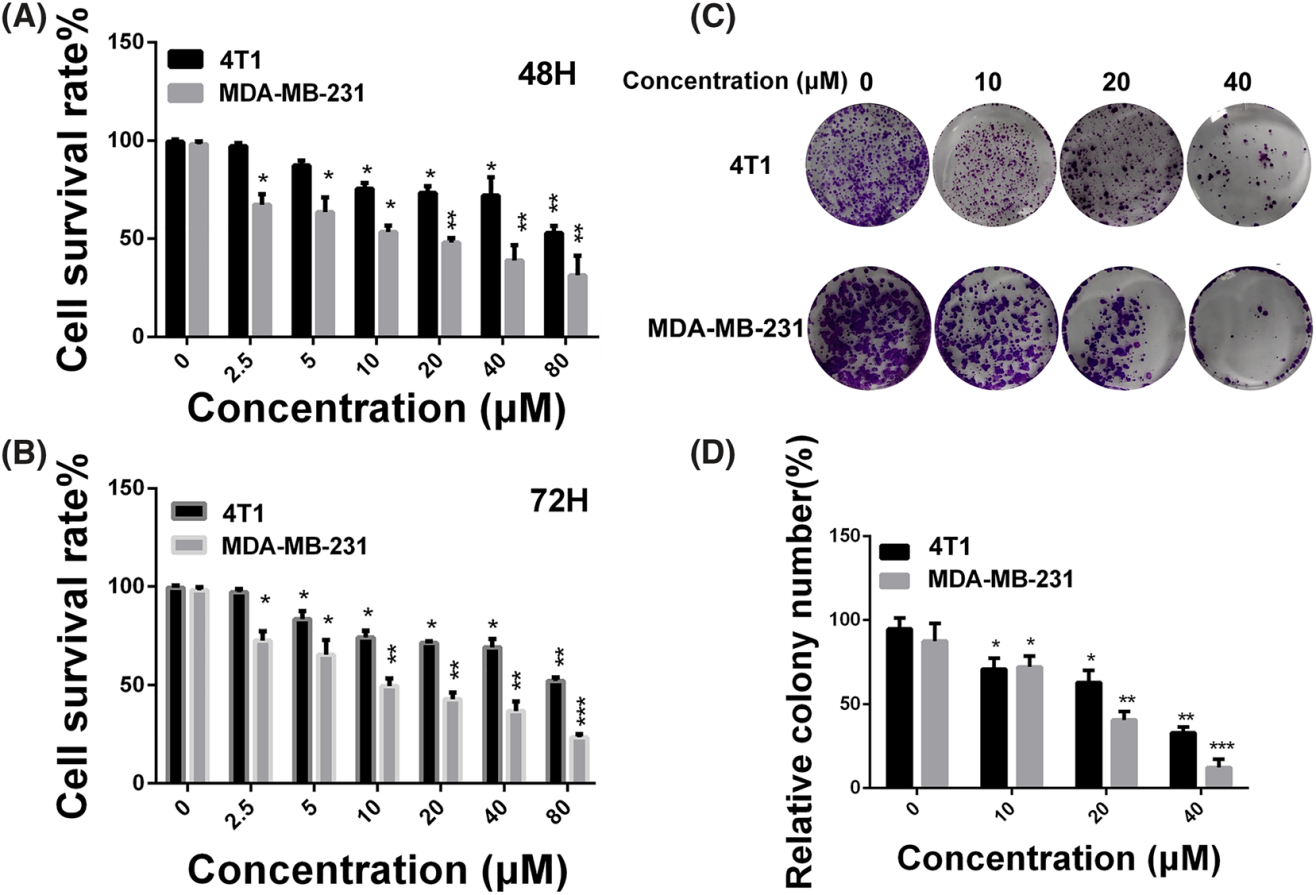
Anti‐proliferation effect of hinokiflavone in breast cancer cells. (A and B) MDA‐MB‐231 and 4T1 cells were treated with different concentrations of hinokiflavone for 48 and 72h. Cell viability was determined by using the MTT method. (C and D) The effects of hinokiflavone on colony formation in MDA‐MB‐231 cells for 9 days. Data are displayed as means ± SD from three independent experiments

We exploited MTT assay to test anti‐proliferation effect of hinokiflavone on MDA‐MB‐231 and 4T1 cell lines. As shown in Figures 2A and 2B, after treated with different concentrations of hinokiflavone for 48h and 72h, cell viability of both MDA‐MB‐231 and 4T1 were significantly inhibited. Hinokiflavone exhibited a time‐ and concentration‐dependent anti‐proliferation effect in MDA‐MB‐231 and 4T1 cells.

Colony formation assay was performed to further assess the anti‐proliferation effect of hinokiflavone. As shown in Figures 2C and 2D, the colony formation of melanoma cells was inhibited with the increase of hinokiflavone concentration after 24 hours treatment. In addition, the colony size was significantly smaller than that of the control group. Taken together, these results suggest that hinokiflavone could effectively suppress breast cancer cells proliferation.

### 3.2 Hinokiflavone induces apoptosis in breast cancer cells

**Figure 3:**
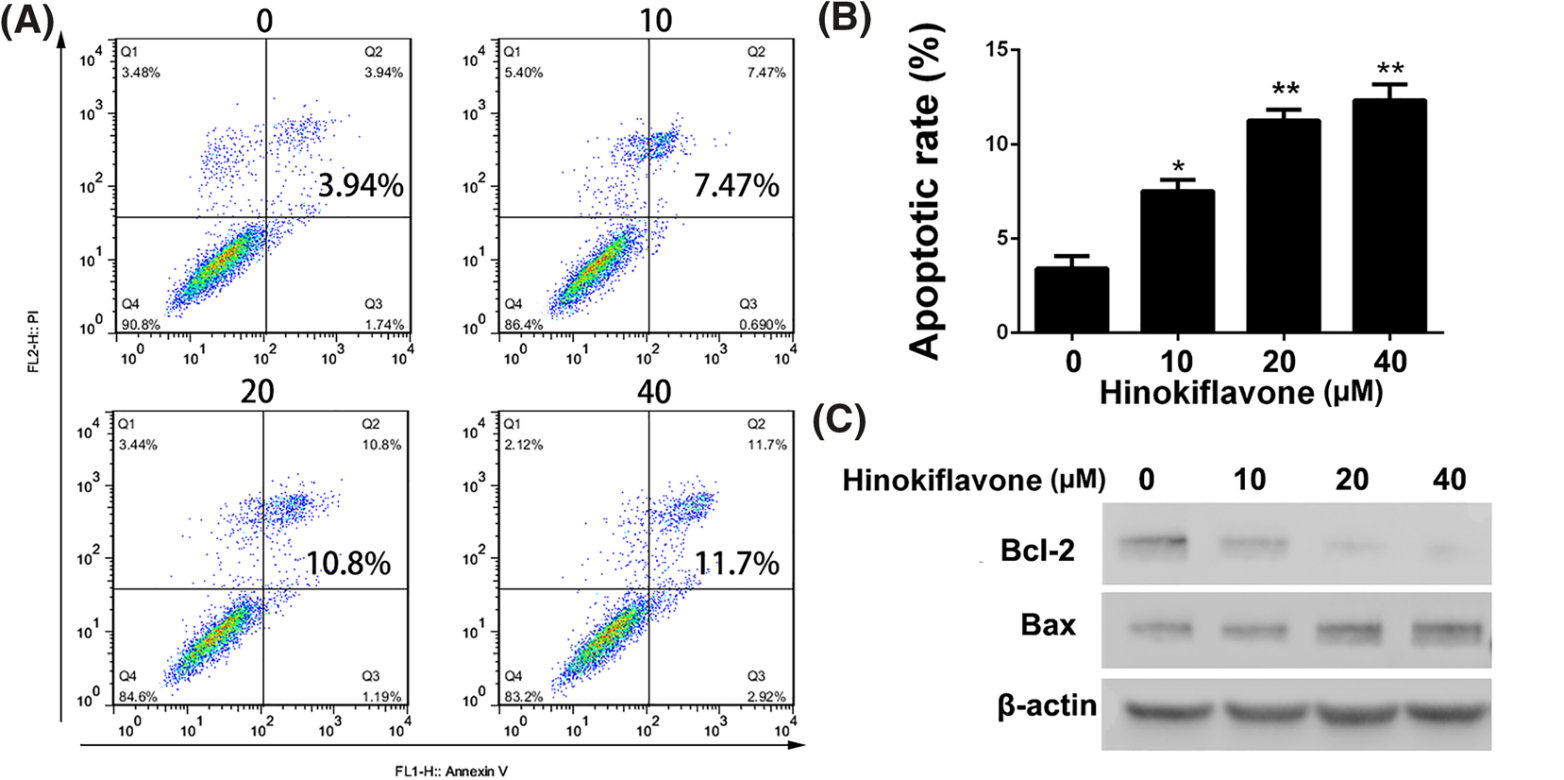
Apoptosis induction of hinokiflavone in breast cancer cells. (A and B) MDA‐MB‐231 cells were treated with different concentrations of hinokiflavone for 24h. Apoptotic cells were stained with Annexin V‐FITC/PI and tested by FCM. (C) Western blot analysis of apoptosis associated proteins after treatment with hinokiflavone. Data are displayed as means ± SD from three independent experiments

Cell apoptosis induction by hinokiflavone was evaluated by Annexin V‐FITC/PI dual‐labelling technique and the levels of apoptosis were investigated by FCM. As shown in Figures 3A and 3B, compared with control group, treatment of 40 μM of hinokiflavone significantly induced highest cell apoptotic rate (11.7± 3.2%, *P*<0.005). Importantly, we can find that the apoptotic rates in MDA‐MB‐231 cells were significantly increased as the concentration of hinokiflavone increased.

In addition, apoptosis‐related proteins were analysed by western blot. The expression level of Bax and Bcl‐2 is displayed in Figure 3C. The expression level of Bcl‐2, which is regarded as an anti‐apoptotic protein, was dramatically inhibited while the expression level of Bax was upregulated with a dose‐dependent manner. These results indicated that hinokiflavone probably induce apoptosis in breast cancer cells via mitochondrial apoptosis pathway.

### 3.3 Hinokiflavone suppresses migration and invasion in breast cancer cells via EMT signalling pathway

**Figure 4:**
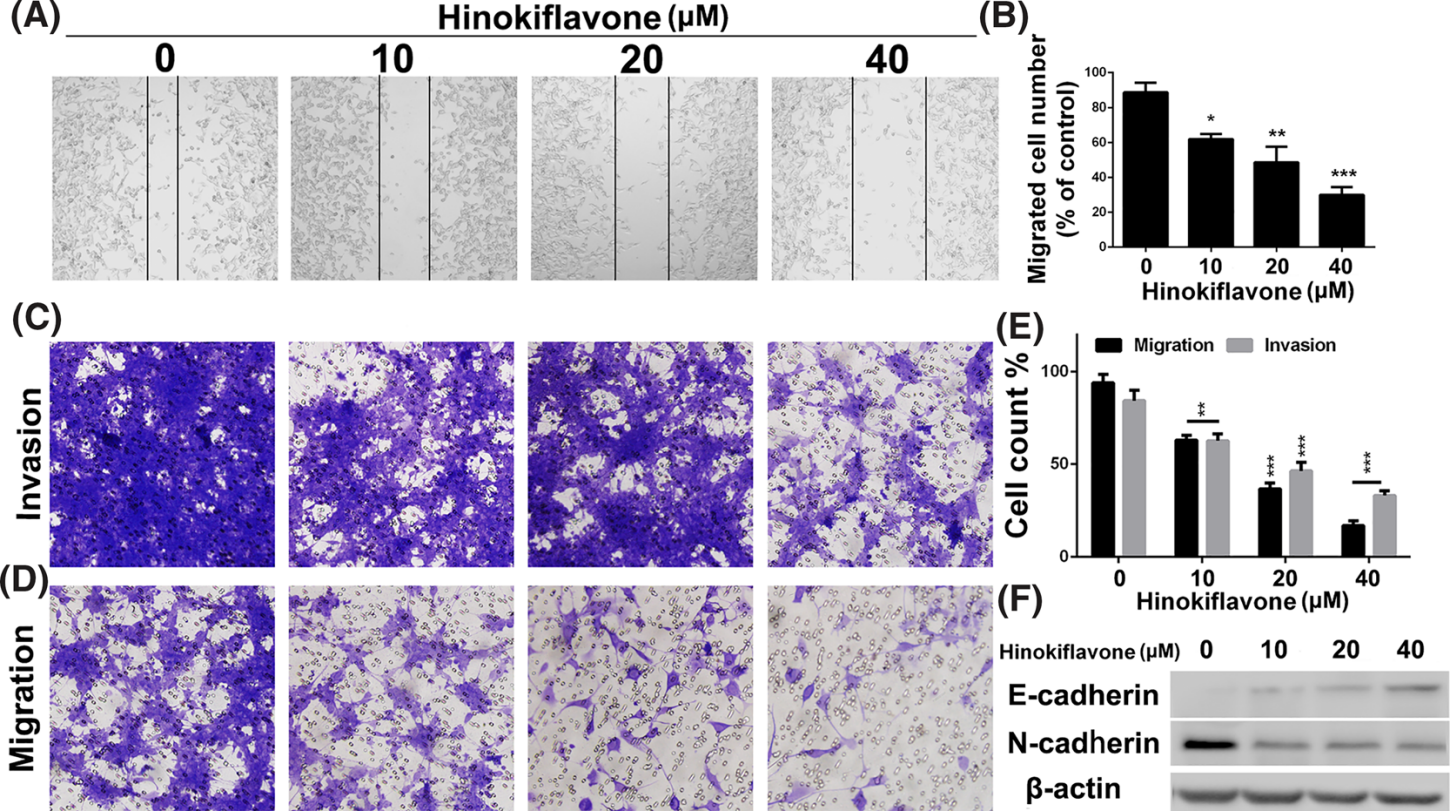
Anti‐migration and anti‐invasion evaluation of hinokiflavone in breast cancer cells. (A and B) MDA‐MB‐231 cells were seeded into 6‐well plates. A single scratch was made when cells grew about 80% confluence. When treated with hinokiflavone for 24 h, cells were fixed and imaged. The lines indicate the area of migrated cells which were quantified. (C, D and E) Transwell migration and invasion evaluation of MDA‐MB‐231 cells after treatment with different concentrations of hinokiflavone for 48 h. (F) Western blot analysis of EMT associated proteins after treatment with different concentrations of hinokiflavone for 24 h. Data are displayed as means ± SD from three independent experiments

The migration and invasion of tumour cells are critical for the process of tumour metastasis.^23^ In addition, considerable metastatic capacity accounts for the high mortality of breast cancer.^24^, ^25^ Therefore, we exploited the wound healing assays and transwell assays to evaluate whether hinokiflavone possess a blocked effect on migration and invasion in breast cancer cells. As displayed in Figures 4A and 4B, cells, which were treated with different concentrations of hinokiflavone, exhibited weak migration ability. Moreover, hinokiflavone showed significantly anti‐migration and anti‐invasion effect by a dose‐dependent manner in comparison with control group (*P*<0.001, Figures 4C–4E).

Epithelial‐to‐mesenchymal transition (EMT) is a cellular program which responsible for tumour cells metastasis. EMT enables tumour cells to gain the features of highly malignant proliferation, notably motility and overt invasiveness.^26^ Hence, we investigated whether the expression level of EMT associated proteins were influenced by treatment of hinokiflavone. As shown in Figure 4F, we found that hinokiflavone could significantly up‐regulated the expression level of E‐cadherin, and down‐regulated N‐cadherin of MDA‐MB‐231 cells in a concentration‐dependent manner. These results suggested that hinokiflavone has potent ability to inhibit breast cancer cell migration and invasion via the EMT signalling pathway.

### 3.4 **Antitumour efficacy of hinokiflavone in a** tumour **xenograft model of breast cancer cells**

**Figure 5:**
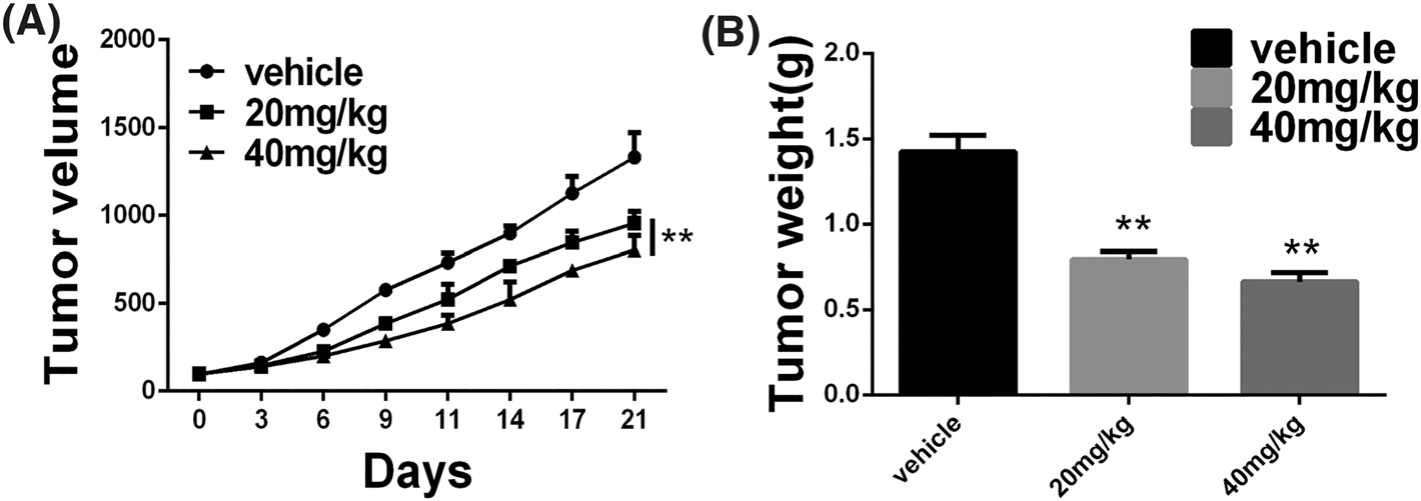
Anti‐tumour evaluation of hinokiflavone *in vivo*. (A) Growth curve of MDA‐MB‐231 cells xenograft tumours was drawn by measuring tumour volumes at determined days. (B) Tumour weight of different treatment groups at 21 days after inoculation. Data are displayed as means ± SD from three independent experiments

To explore whether the antitumour activity of hinokiflavone *in vivo* is consistent with its effects in vitro, MDA‐MB‐231 bearing mice were dosed daily at the designated doses (control, 20 and 40 mg kg^−1^) for 21 days. As a key indicator of health, the average body weight of the control (corn oil treated) and hinokiflavone‐treated mice did not differ significantly throughout the experiment (data not shown). As displayed in Figure 5A, tumour growth was significantly inhibited by treatment of hinokiflavone. Hinokiflavone distinctly reduced tumour weight in a dose‐dependent manner (Figure 5B).

**Figure 6:**
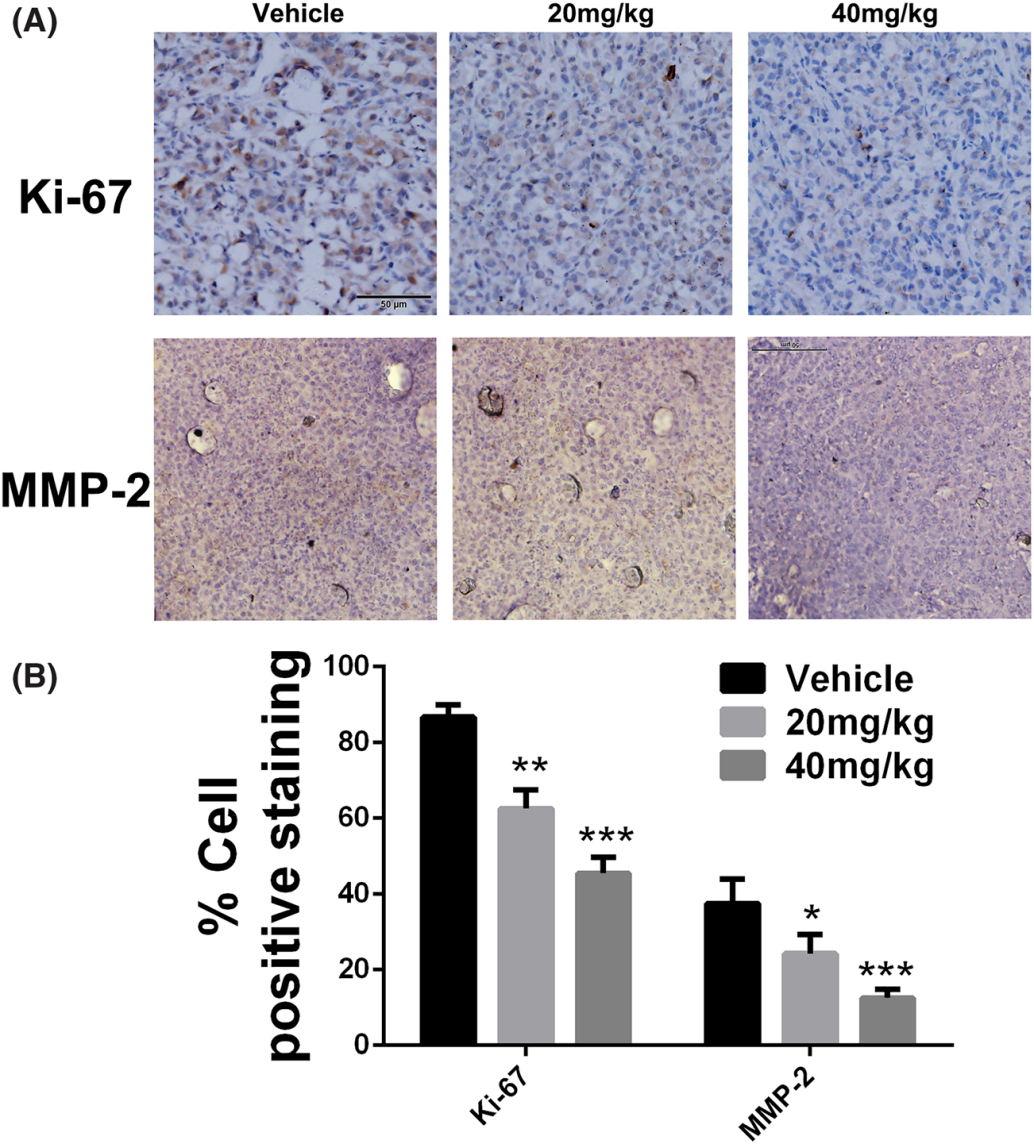
Immunohistochemical analysis of anti‐proliferation and anti‐metastasis of hinokiflavone. (A) Immunohistochemical analysis of Ki‐67 and MMP‐2 of tumour sections after different treatments. (B) Quantitation of Ki‐67^+^ and MMP‐2^+^ cells. Data are displayed as means ± SD from three independent experiments

As evident from the results of immune histochemistry staining, less Ki67‐positive and MMP‐2‐positive cells were observed in the tumours sections from hinokiflavone‐treated mice compared with the control mice (Figures 6A and 6B), indicating that hinokiflavone impedes human breast cancer cells growth *in vivo*, which is consistent with the findings *in vitro*.

## 4 DISCUSSIONS

Breast cancer is the most prevalent cancer among women worldwide and the second most common cause of cancer death in women.^1^ Even though dramatic advances have been made in screening methods and treatments of breast cancer over the last decade, the overall survival of patients with breast cancer is still quite low due to the high rate of vital organ metastasis. Therefore, it is of great need to develop novel diagnostic and therapeutic agents to improve the treatment of breast cancer. Although the chemical drugs have a greater destruction of tumour cells but also a strong toxic side effect. Natural extracts derived from plants and animals have the features of high efficiency and low toxicity. Previous studies have shown that hinokiflavone is cytotoxic to different types of human cancer cells. In this study, we assessed the anti‐cancer activity of hinokiflavone in human and mouse breast cancer cells *in vitro* and *in vivo*.

We first performed MTT and colony formation experiments to investigate the anti‐proliferation effect of hinokiflavone in vitro. The results showed that hinokiflavone had strong cytotoxic and inhibitory effects on breast cancer cells. To further investigate the anti‐proliferation mechanism of hinokiflavone in breast cancer cells, FCM analysis was performed to assess the effect of hinokiflavone on apoptosis of MDA‐MB‐231 cells. Additionally, expression level of Bax and Bcl‐2, two key apoptosis‐related proteins were analysed. The results showed that the expression levels of Bax was significantly upregulated, while the expression of Bcl‐2 protein was reduced, which suggesting that hinokiflavone probably induce apoptosis in breast cancer cells via mitochondrial apoptosis pathway.^27^, ^28^ For the purpose of investigating the effect of hinokiflavone on the metastatic ability of breast cancer cells, we conducted transwell migration and invasion experiments. We found that hinokiflavone could obviously suppress the migration and invasion ability of MDA‐MB‐231 cells by impairing process of EMT. It is reported that EMT are associated with the acquisition of aggressive and invasive phenotypes in various cancer cells and regarded as an essential process for tumour metastasis.^27^, ^28^, ^29^, ^30^ Moreover, results of *in vivo* experiments have shown that hinokiflavone can suppress tumour growth by inhibiting cells proliferation and metastasis, which is consistent with the findings of *in vitro* experiments.

In summary, we evaluated the anti‐cancer activity of hinokiflavone in breast cancer cells by *in vitro* and *in vivo* experiments. Hinokiflavone significantly inhibited proliferation and induced apoptosis in breast cancer cells. In addition, our results showed that hinokiflavone remarkably inhibited migration and invasion of breast cancer cells via EMT signalling pathway. It is worth noting that hinokiflavone possesses anti‐tumour effect in tumour mouse xenograft model of breast cancer. Overall, our results indicated that hinokiflavone may be a potential anticancer drug for breast cancer treatment.

## AVAILABILITY OF DATA AND MATERIALS

The data sets used or analysed in this study are available from the corresponding author on reasonable request.

## AUTHORS' CONTRIBUTIONS

XY and ZG designed the study. XY and CL wrote the article. XY, CL, WX and CL collected and analysed the data. SL and ZG collected and analysed the data to revise the manuscript in accordance with reviewer's comments. Also, SS helped the authors write the revised version. All authors read and approved the final manuscript.

## ETHICS APPROVAL AND CONSENT TO PARTICIPATE

All of the animal experiments in this study were performed according to the National Institutes of Health (Bethesda, MD, USA) guidelines and were approved by the Ethical Committee of First Affiliated Hospital of Gannan Medical University (Ganzhou, China).

## CONSENT FOR PUBLICATION

Consent for the publication of the clinical and pathological data was obtained from all patients who were involved in this study.

## FUNDING

No funding was received.

## CONFLICT OF INTEREST

The authors have declared that there is no conflict of interest.
